# An interpretable Alzheimer’s disease oligogenic risk score informed by neuroimaging biomarkers improves risk prediction and stratification

**DOI:** 10.3389/fnagi.2023.1281748

**Published:** 2023-10-26

**Authors:** Erica H. Suh, Garam Lee, Sang-Hyuk Jung, Zixuan Wen, Jingxuan Bao, Kwangsik Nho, Heng Huang, Christos Davatzikos, Andrew J. Saykin, Paul M. Thompson, Li Shen, Dokyoon Kim, for the Alzheimer’s Disease Neuroimaging Initiative

**Affiliations:** ^1^Department of Biostatistics, Epidemiology and Informatics, Perelman School of Medicine, University of Pennsylvania, Philadelphia, PA, United States; ^2^Innovative Medical Technology Research Institute, Seoul National University Hospital, Seoul, Republic of Korea; ^3^Department of Radiology and Imaging Sciences, School of Medicine, Indiana University School of Medicine, Indianapolis, IN, United States; ^4^Department of Biomedical Informatics, University of Pittsburgh, Pittsburgh, PA, United States; ^5^Center for Biomedical Image Computing and Analytics, Department of Radiology, University of Pennsylvania, Philadelphia, PA, United States; ^6^Imaging Genetics Center, Mark and Mary Stevens Neuroimaging and Informatics Institute, Keck School of Medicine, University of Southern California, Los Angeles, CA, United States; ^7^Institute for Biomedical Informatics, University of Pennsylvania, Philadelphia, PA, United States

**Keywords:** polygenic risk score, Alzheimer’s disease, mild cognitive impairment, genetics, predictive markers

## Abstract

**Introduction:**

Stratification of Alzheimer’s disease (AD) patients into risk subgroups using Polygenic Risk Scores (PRS) presents novel opportunities for the development of clinical trials and disease-modifying therapies. However, the heterogeneous nature of AD continues to pose significant challenges for the clinical broadscale use of PRS. PRS remains unfit in demonstrating sufficient accuracy in risk prediction, particularly for individuals with mild cognitive impairment (MCI), and in allowing feasible interpretation of specific genes or SNPs contributing to disease risk. We propose adORS, a novel oligogenic risk score for AD, to better predict risk of disease by using an optimized list of relevant genetic risk factors.

**Methods:**

Using whole genome sequencing data from the Alzheimer’s Disease Neuroimaging Initiative (ADNI) cohort (*n*  =  1,545), we selected 20 genes that exhibited the strongest correlations with FDG-PET and AV45-PET, recognized neuroimaging biomarkers that detect functional brain changes in AD. This subset of genes was incorporated into adORS to assess, in comparison to PRS, the prediction accuracy of CN vs. AD classification and MCI conversion prediction, risk stratification of the ADNI cohort, and interpretability of the genetic information included in the scores.

**Results:**

adORS improved AUC scores over PRS in both CN vs. AD classification and MCI conversion prediction. The oligogenic model also refined risk-based stratification, even without the assistance of APOE, thus reflecting the true prevalence rate of the ADNI cohort compared to PRS. Interpretation analysis shows that genes included in adORS, such as ATF6, EFCAB11, ING5, SIK3, and CD46, have been observed in similar neurodegenerative disorders and/or are supported by AD-related literature.

**Discussion:**

Compared to conventional PRS, adORS may prove to be a more appropriate choice of differentiating patients into high or low genetic risk of AD in clinical studies or settings. Additionally, the ability to interpret specific genetic information allows the focus to be shifted from general relative risk based on a given population to the information that adORS can provide for a single individual, thus permitting the possibility of personalized treatments for AD.

## 1. Introduction

Alzheimer’s disease (AD) is the most common cause of dementia. It is an irreversible, progressive neurodegenerative disorder characterized by abnormal accumulation of amyloid plaques and neurofibrillary tangles in the brain, and disruption of memory, thinking, and behavior (Mullard, 2021). Because the root cause of AD remains unclear, current treatments only temporarily inhibit cognitive symptoms and fail to directly modify the course of the disease (Zetterberg and Bendlin, 2021). Limited progress of therapeutic development in clinical trials urges the need to consider alternative strategies for AD treatments, namely by investigating patients who are at risk of developing AD before disease onset.

Mild cognitive impairment (MCI)—a transitional stage between cognitively normal (CN) and early AD patients—may enable earlier detection of AD pathogenesis before clinical diagnosis (Alzheimer Association, 2021). Many investigators regard this phase as critical, for substantial neuronal damage has already occurred by the time the degenerative process of AD has fully developed. 15% of MCI individuals develop dementia after 2 years and 32% progress to AD within 5 years’ follow-up, while others remain cognitively normal (Ward et al., 2013). The ability to predict progression from MCI to AD is critical for timely treatment to prevent or delay cognitive decline.

Polygenic risk scores (PRS) are a common approach for stratifying patients into levels of risk for AD by calculating the weighted sum of GWAS-significant SNP genotypes (Baker and Escott-Price, 2020; Sims et al., 2020; Leonenko et al., 2021; Gouveia et al., 2022). By considering the cumulative effects of many genetic variants together, one can better predict an individual’s risk for a disease compared to considering single variants in isolation. Although AD’s delayed symptom onset makes it difficult to manage, a deeper grasp of the underlying biology and early risk detection may pave the way for improved treatments for AD. This has, in part, motivated the application of PRS for AD (Marden et al., 2016; Desikan et al., 2017; Harrison et al., 2020; Leonenko et al., 2021; Jung et al., 2022). Previous studies have shown that PRS are able to quantify differences in genetic risk between individuals, enable risk-based stratification, and demonstrate strong associations with known markers of neurodegeneration (Desikan et al., 2017; Tan et al., 2019; Huq et al., 2021; Leonenko et al., 2021). However, despite longstanding evidence that AD risk is polygenic (Altmann et al., 2020), more recent studies have proposed that AD may in fact be an oligogenic disorder (Zhang et al., 2020). This leaves an open-ended discussion for how genetic risk for AD must be represented.

One primary limitation of PRS is that its calculation relies on genome-wide association studies (GWAS) of AD case–control datasets, which often use clinical diagnosis as phenotypes. However, AD clinical diagnosis partially relies on cognitive assessment outcomes, which may be influenced by factors unrelated to the disease such as the patient’s anxiety, fatigue, and general test-taking ability. Thus, it remains a challenge to identify genetic risk factors for AD solely using case–control-based GWASs. Additionally, GWAS studies examining MCI to AD progression indicate that AD susceptibility loci exhibit minimal effects. Current datasets with MCI individuals may lack the statistical power to identify significant SNPs, particularly with the inclusion of MCI subjects unlikely to develop AD or those progressing to other dementia types. Consequently, the observed effect sizes of true AD susceptibility genes in these MCI cohorts are further diminished compared to traditional AD case–control datasets, undermining the utility of PRS for individuals with MCI (Lacour et al., 2017; Chaudhury et al., 2019). Furthermore, PRS is not advocated for clinical use, as it not only is prone to bias, but also is unable to demonstrate sufficient specificity and sensitivity in risk prediction (Cuyvers and Sleegers, 2016).

Conversely, neuroimaging data, also commonly used for AD diagnosis, e.g., magnetic resonance imaging (MRI), 2-Deoxy-2-[^18^F] fluorodeoxyglucose (FDG)-PET, and [^18^F] Florbetapir (AV45)-PET, are a highly reproducible and more objective data source (Sperling et al., 2011; Suppiah et al., 2019). FDG-PET tracks glucose metabolism and is particularly useful for identifying areas of the brain distinctly impacted by AD, although its accuracy may decline in older patients (Foster et al., 2007; Sperling et al., 2011). AV45-PET specifically visualizes amyloid-beta plaques, a key hallmark of AD, offering a significant advancement over previous methods that could only confirm these plaques post-mortem (Suppiah et al., 2019). Compared to other diagnostic measures like cerebrospinal fluid (CSF) tests and cognitive assessments, these PET imaging techniques are offer a real-time view of critical pathological features in the brain.

Recent studies have demonstrated that neuroimaging biomarkers can effectively predict AD progression. Follow-up data of AD patients exhibited reduced FDG uptake in the frontal, parietal and lateral temporal lobes, indicating strong biomarkers for MCI conversion prediction (Ossenkoppele et al., 2012; Caminiti et al., 2018). Huang et al. (2017) used longitudinal MRI data to classify MCI subjects as either MCI converters (MCI-c) or MCI non-converters (MCI-nc), achieving 79.4% accuracy. Integrating MRI with FDG-PET data increased prediction performance to 86.4% (Lu et al., 2018; Lee et al., 2019). These findings demonstrate that neuroimaging endophenotypes have the potential to reflect genotypes correlated with brain structure and function and may also help characterize the mechanisms of such risk alleles or genes associated with AD risk. Identifying SNPs or genes that affect or are correlated with changes in neuroimaging traits can further our knowledge of the genetic underpinnings of AD risk and progression.

As such, we generated adORS, a novel oligogenic risk score for Alzheimer’s disease, to robustly predict high or low risk of disease and the likelihood of MCI-to-AD conversion. This risk score leverages neuroimaging biomarkers FDG-PET and AV45-PET (Shen and Thompson, 2020) to extract a smaller, more informative set of AD-associated genes to ultimately improve risk prediction performance for two separate tasks: CN vs. AD classification (CNAD) and MCI conversion prediction (MCI-CP). We compared prediction performance between adORS and conventional PRS and interpreted the genetic contributions within each score.

## 2. Materials and methods

### 2.1. ADNI and ADSP-FUS1-ADNI-WGS-2 cohorts

Alzheimer’s Disease Neuroimaging Initiative (ADNI; ADNI-WGS-1, 2020) is a multisite longitudinal study that tracks the progression of AD in the human brain with clinical, imaging, genetic and biospecimen biomarkers through the process of normal aging, early mild cognitive impairment, and late mild cognitive impairment to dementia or AD. The overall goal of ADNI is to validate biomarkers for use in Alzheimer’s disease clinical treatment trials. ADSP-FUS1-ADNI-WGS-2 indicates an additional set of ADNI participants that underwent whole genome sequencing (WGS) as a collaboration between ADNI and ADSP Follow-Up Study (FUS; ADSP FUS1 WGS, 2021). We focus on the use of WGS data (see section 2.2) and baseline FDG-PET and AV45-PET biomarkers (see section 2.3), which are inherently cross-sectional. Hence, this a cross-sectional study. We collected data from 1,545 individuals across all modalities.

In the ADNI study, each participant receives one of four diagnoses during each visit: CN, early MCI, late MCI, or AD. Follow up data post baseline occurs every 6 months and average about 5 years in duration. To transform the longitudinal diagnoses into a cross-sectional format, we assigned each individual with a single, cumulative label based on their diagnosis history from all available visits. The labels are as follows for the two separate tasks:

1. CNAD task: AD cases (AD) and cognitively normal controls (CN).
2. MCI-CP task: MCI converters (MCI-c) and MCI non-converters (MCI-nc).

We generated these definitions for each label:

- MCI-c: Diagnosed with early or late MCI at baseline and later progresses to AD, maintaining this until the last visit.
- MCI-nc: Diagnosed with early or late MCI at baseline and remains with this diagnosis through the final visit.
- AD: Either consistently diagnosed with AD or categorized as MCI-c.
- CN: Remains cognitively normal from the first to the last visit.

Using these definitions, we acquired 550 AD cases, 364 CN controls, 273 MCI converters, and 517 MCI non-converters (Table 1).

**Table 1:** ADNI and ADSP-FUS1-ADNI-WGS-2 data demographics.

	Cohort (top: control, bottom: case)	Cohort definition	*N* samples	Mean age (SD)	% Males/Females	APOE distribution (ε2ε2/ε2ε3/ε2ε4/ε3ε3/ε3ε4/ε4ε4)	Mean cognitive test scores (SD)				
							ADAS13	CDRSB	FAQ	MMSE	RAVLT
CN vs. AD classification	Cognitive normal	CN diagnosis from first to last visit	364	74.69 (7.47)	50.82/49.18	2/46/2/214/89/10	9.34 (4.34)	0.03 (0.13)	0.17 (0.63)	29.06 (1.15)	5.81 (2.28)
	Alzheimer’s disease	CN or AD diagnosis at first visit, AD at last visit	550	74.32 (5.74)	58.91/41.09	1/17/10/174/252/96	25.55 (8.56)	3.19 (1.87)	9.48 (7.26)	25.03 (2.74)	2.43 (2.08)
MCI conversion prediction	MCI non-converters	EMCI or LMCI diagnosis from first to last visit	517	72.48 (7.81)	58.41/41.59	1/37/14/263/159/42	14.07 (5.89)	1.28 (0.73)	1.92 (3.06)	28.01 (1.69)	4.72 (2.54)
	MCI converters	EMCI or LMCI diagnosis at first visit, AD at last visit	273	73.84 (7.01)	62.27/37.73	0/8/6/92/124/43	20.91 (6.20)	1.90 (0.98)	5.38 (4.85)	26.97 (1.77)	2.99 (2.22)

Phenotypic information available in ADNI for each classification problem: cohort cases and controls, criteria used to define cases and controls, number of samples, mean age and standard deviations, APOE distribution, and mean cognitive scores and standard deviations (see Section 2.7 for details on cognitive scores). CN, Cognitively normal; AD, Alzheimer’s disease; MCI, Mild cognitive impairment; EMCI, Early mild cognitive impairment; LMCI, Late mild cognitive impairment.

### 2.2. WGS genotyping and quality control

A total of 1,566 samples were genotyped from ADNI and ADNI-WGS-2 with ADSP Follow-Up Study. WGS genotyping was performed using HiSeq2000; Read length (bp) 100; genome assembly GRCh38 (hg38). Quality Control {QC; SNV concordance check, sex mismatch, contamination check, and relatedness [(Pihat >0.4)]} were performed primarily using the SNP/Indel Variant Calling Pipeline (VCPA) v1.0 developed and maintained by National Institute on Aging Genetics of Alzheimer’s Disease Data Storage Site (NIAGADS; Leung et al., 2019). All QC quality checks were implemented by the Genome Center for Alzheimer’s Disease (GCAD) Data Production Team. And variants were filtered out with low-quality, multi-allelic, and monomorphic SNVs using filters of GQ < 20 and DP < 10 and a missing rate > 20% and a *p* < 1.0 × 10^−6^ for the Hardy–Weinberg equilibrium test. After QC, 15,456,635 variants and 1,545 samples remained available for performing PRS and adORS in the ADNI and ADSP-FUS1-ADNI-WGS-2 datasets.

### 2.3. FDG-PET and AV45-PET

Every FDG and AV45 PET scan is reviewed for protocol compliance by the ADNI PET QC team. PET measures are reported as standard uptake value ratios (SUVRs), which is a quantification of the radiotracer concentration within specific region of interests (ROI) in the body (ADNI PET analysis method, 2023). The average AV45 SUVR was computed from regions including the frontal, anterior cingulate, precuneus, and parietal cortex relative to the cerebellum. For FDG-PET, the average SUVR was based on the uptake in the angular, temporal, and posterior cingulate regions, areas known to exhibit altered metabolic activity in Alzheimer’s disease. Such measures help elucidate the metabolic changes associated with the disease’s progression.

### 2.4. PRS calculation

To calculate PRS, we used summary statistics from a large-scale GWAS study done for AD on the International Genomics of Alzheimer’s Project (IGAP) consortium to generate genetic scores for ADNI participants. The IGAP GWAS was re-performed excluding 441 ADNI participants (55,931 participants remained across the Alzheimer’s Disease Genetic Consortium (ADGC), the Cohorts for Heart and Aging Research in Genomic Epidemiology (CHARGE) Consortium, the European Alzheimer’s Disease Initiative (EADI), and the Genetic and Environmental Risk in Alzheimer’s Disease (GERAD) Consortium). We generated PRS with the PLINK genetic data analysis toolset using clumping and thresholding (C + T), a common method which removes redundantly correlated variants and preferentially retains those most associated with the phenotype of interest. This method was chosen based on the suggested method of PRS calculation and threshold cutoffs performed in Leonenko et al. (2021). In our calculations, SNPs in the APOE region were first removed (chr19:44.0–46.0 Mb, GRCh38), then clumped by excluding nearby variants within a 1,000-kb window with *r*^2^ > 0.1, and finally filtered using the threshold pT ≤ 1e−5. We also computed additional PRSs using pT ≤ 5e−8, 0.1, 0.5 (Supplementary Table 1). Given that SNPs in the APOE region were removed from the PRS calculation, we modeled APOE separately as the weighted sum of alleles ε2 and ε4 using effect sizes found in the IGAP summary statistics [β(ε2) = −0.47, β(ε4) = 1.12].

### 2.5. adORS calculation

Our proposed method is comprised of three steps for adORS calculation: gene-level aggregation, gene selection, and model training. First, SNPs of each patient were aggregated into their corresponding gene burdens. It is denoted in Equations (1, 2) and where $v_{ij}$ represents j -th gene burden of patient i . $s_{ik}$ in Equation (1) is the transformation of k -th variant:


(1) $$s_{ik}=\{\begin{array}{c} \;2-x_{ik},ifnegativeassociationwith\;AD\\ x_{ik},otherwise\; \end{array}$$

(2) $$v_{ij}=\overset{g_{j}}{\underset{k}{\sum }}s_{ik}$$

Gene burdens were simply computed by summing each SNP or complement of SNP in a given gene depending on the direction of each variant’s effect on Alzheimer’s disease. For example, a variant is summed after complement operation if the variant is negatively associated with the AD while regular variant is added otherwise. This prevents variants in opposite direction from canceling out each variants’ effects. Genes were defined using refGene database and the ANNOVAR tool. Second, gene selection is performed to condense the number of genes included in calculating adORS. We measured Pearson correlation coefficients with each neuroimaging biomarker, FDG-PET and AV45-PET, and then, extracted the top 20 genes exhibiting the highest correlation (Supplementary Figure 1; Supplementary Table 2). Since FDG-PET and AV45-PET have been known as promising biomarkers to detect functional brain changes in AD, we used them as intermediate phenotypes to discover AD-relevant genes (Foster et al., 2007; Sperling et al., 2011; Suppiah et al., 2019). After accounting for overlapping genes from each set of 20 genes, the total of 37 genes were used to calculate adORS. Finally, the risk score for each patient 𝑖 was estimated based on the final condensed gene set 𝐺:


(3) $$adORS_{i}=\overset{G}{\underset{j}{\sum }}\beta_{j}v_{ij}$$

$\beta_{j}$ in Equation (3) is the weight associated with each gene burden $v_{ij}$ , calculated by a logistic regression (LR) model trained on the task of interest. Given that we focus on two problems, CNAD and MCI-CP, a separate LR model was trained for each task. As mentioned in section 2.1, only CN and AD subjects were used to train the CNAD LR model, and correspondingly, only MCI-nc and MCI-c subjects were used to train the MCI-CP model. Each LR model was implemented from the sklearn package in Python and adjusted for age and gender. They were developed and assessed using an 80:20 train test split and 10-fold cross-validation, with each fold containing the same ratio of positive and negative samples from the ADNI cohort. Prediction accuracy of the PRS and adORS models was measured using the Area Under Receiver Operating Characteristic (AUROC) and Area Under the Precision-Recall Curve (AURPC).

### 2.6. Gene contribution

Gene contribution of adORS was calculated using Shapley Additive exPlanations (SHAP; Lundberg and Lee, 2017). SHAP is a model-agnostic interpretation method frequently used in deep neural network and tree-based ensemble models. The strategy of SHAP is to measure features’ relative effect contributing to model’s output while considering not only isolated effect of a single feature but also interactions between features. We applied SHAP package to trained adORS model to generate feature importance, namely SHAP value. To calculate gene contribution of PRS, we transformed variant-level into gene-level contribution. Since there is no gene-level summary statistics in PRS, we first calculated variant-level contribution using summary statistics multiplied by variant ( $\beta_{k}x_{ik}$ ), and then the measures of variants’ contribution in a given gene were summed. Instead of investigating coefficient ( $\beta_{k}$ ), we defined $\beta_{k}x_{ik}$ as a contribution measure of k -th variants because it not only reflects actual contribution to the level of risk score, but also enables to compute gene-level feature importance.

### 2.7. Phenotype association

Five scores (ADAS13, CDRSB, RAVLT, MMSE, and FAP) assessing the abilities of orientation to place and time, language, reasoning, and the subjects’ daily living activities were downloaded from Quantitative Templates for the Progression of Alzheimer’s disease (QT-PAD) challenge (Portland Institute for Computational Science, 2022). We preserved the baseline visit for each subject (Table 1). We matched the subjects in our adORS analysis and QT-PAD baseline visit. Subjects without any of the five cognitive scores were excluded. The training and testing subjects are matched with our adORS analysis. We calculated Pearson correlations between adORS/PRS and five cognitive function assessment scores.

## 3. Results

### 3.1. adORS vs. PRS model performance comparison

To evaluate adORS’s ability to predict AD status and progression, we compared classification performance of adORS to PRS using the following evaluation metrics: Area Under Receiver Operating Characteristic (AUROC) and Area Under the Precision-Recall Curve (AURPC). Performance was compared on two tasks: CN vs. AD classification and MCI conversion prediction. Table 2 details the description of each model. The PRS and adORS models were computed excluding SNPs in the *APOE* region (see section 2). Two additional models were generated by adding *APOE* as a separate independent variable to the feature set. *APOE* effects were modeled by summing the genotyped *APOE* isoforms ε2 and ε4 weighted with GWAS effect sizes from the IGAP summary statistics (Kunkle et al., 2019).

**Table 2:** Descriptions of PRS and adORS models presented in the manuscript.

Model name	Model description
APOE.only	Weighted sum of alleles ε2 and ε4 using effect sizes found in the IGAP summary statistics [β(ε2) = −0.47, β(ε4) = 1.12]
PRS	PRS including SNPs with *p*≤1e-5 and excluding SNPs in the APOE region (chr19:44.0–46.0 Mb, GRCh38)
PRS.with.APOE	PRS including SNPs with *p*≤1e-5 and APOE(ε2 + ε4), where *APOE* effects were weighted with effect sizes [ β (ε2) = −0.47 and β (ε4) = 1.12] from the IGAP summary statistics excluding the ADNI cohort
adORS	Risk score including the top 20 gene burdens with highest correlations to AV45-PET and FDG-PET and excluding the APOE region (chr19:44.0–46.0 Mb, GRCh38)
adORS.with.APOE	Risk score including the top 20 gene burdens with highest correlations to AV45-PET and FDG-PET and APOE(ε2 + ε4), where *APOE* effects were weighted with effect sizes [ β (ε2) = −0.47 and β (ε4) = 1.12] from the IGAP summary statistics excluding the ADNI cohort

As presented in Table 3, adORS_CNAD_ (AUROC = 0.634, AUPRC = 0.717) achieves higher accuracy than PRS_CNAD_ (AUROC = 0.602, AUPRC = 0.694). This suggests that variants used to calculate adORS_CNAD_ hold higher predictive power than those used in PRS. When adding the *APOE* effect to both models, the performances of PRS.with.APOE_CNAD_ and adORS.with.APOE_CNAD_ increase to AUROC = 0.735 and AUROC = 0.750, respectively. The sharp increase in performance from PRS_CNAD_ (AUROC = 0.602) to PRS.with.APOE_CNAD_ (AUROC = 0.735) suggests that the *APOE* loci may be contributing most to prediction, causing other variants to have low effects. While a similar increase in performance was observed from adORS_CNAD_ (AUROC = 0.634) to adORS.with.APOE_CNAD_ (AUROC = 0.750), the higher performance values from the adORS models overall, and in comparison to APOE.only (AUROC = 0.707), demonstrate that additional genes are contributing to AD prediction more than they may be in the PRS models (Table 3). We analyze this phenomenon further in the *Gene contribution* section.

**Table 3:** adORS vs. PRS performance comparisons for CN vs. AD classification and MCI conversion prediction.

Model	AUROC (SD)	AUPRC (SD)
(a) CN vs. AD classification		
APOE.only_CNAD_	0.707 (0.04)	0.746 (0.03)
PRS_CNAD_	0.602 (0.03)	0.694 (0.03)
PRS.with.APOE_CNAD_	0.735 (0.04)	0.814 (0.03)
adORS_CNAD_	0.634 (0.05)	0.717 (0.04)
adORS.with.APOE_CNAD_	0.750 (0.03)	0.815 (0.03)
(b) MCI conversion prediction		
APOE.only_MCI-CP_	0.594 (0.05)	0.417 (0.04)
PRS_MCI-CP_	0.532 (0.04)	0.405 (0.03)
PRS.with.APOE_MCI-CP_	0.643 (0.04)	0.477 (0.04)
adORS_MCI-CP_	0.610 (0.04)	0.464 (0.05)
adORS.with.APOE_MCI-CP_	0.637 (0.04)	0.487 (0.05)

All models were calculated on the ADNI cohort (550 AD cases, 364 CN controls, 273 MCI converters, and 517 MCI non-converters). See the section 2 for details on adORS and PRS calculation. For each task, five models were considered: APOE.only which is the weighted sum of APOE alleles ε2 and ε4 using summary statistics; PRS where the APOE region was excluded (chr19:44.0–46.0 Mb, GRCh38); PRS.with.APOE where APOE effects were weighted with effect sizes as in the summary statistics; adORS which includes the top 20 gene burdens with highest correlations to AV45-PET and FDG-PET; and adORS.with.APOE which also includes weighted APOE effects. Prediction performance was evaluated using AUROC and AUPRC with the standard deviation of 10-fold cross validation.

Similar patterns were observed in the MCI-CP task. Although the performance of PRS_MCI-CP_ was much lower (AUROC = 0.532, AUPRC = 0.405) than in the case of PRS_CNAD_, it comparably increased when the *APOE* region was included (AUROC = 0.643, AUPRC = 0.477). In contrast, only a small increase in performance was observed from adORS_MCI-CP_ (AUROC = 0.610, AUPRC = 0.464) to adORS.with.APOE_MCI-CP_ (AUROC = 0.637, AUPRC = 0.487). This smaller increase in performance in the adORS models further emphasizes the idea that adORS relies less so on the *APOE* loci for the prediction task.

### 3.2. Risk-based stratification

**Figure 1:**
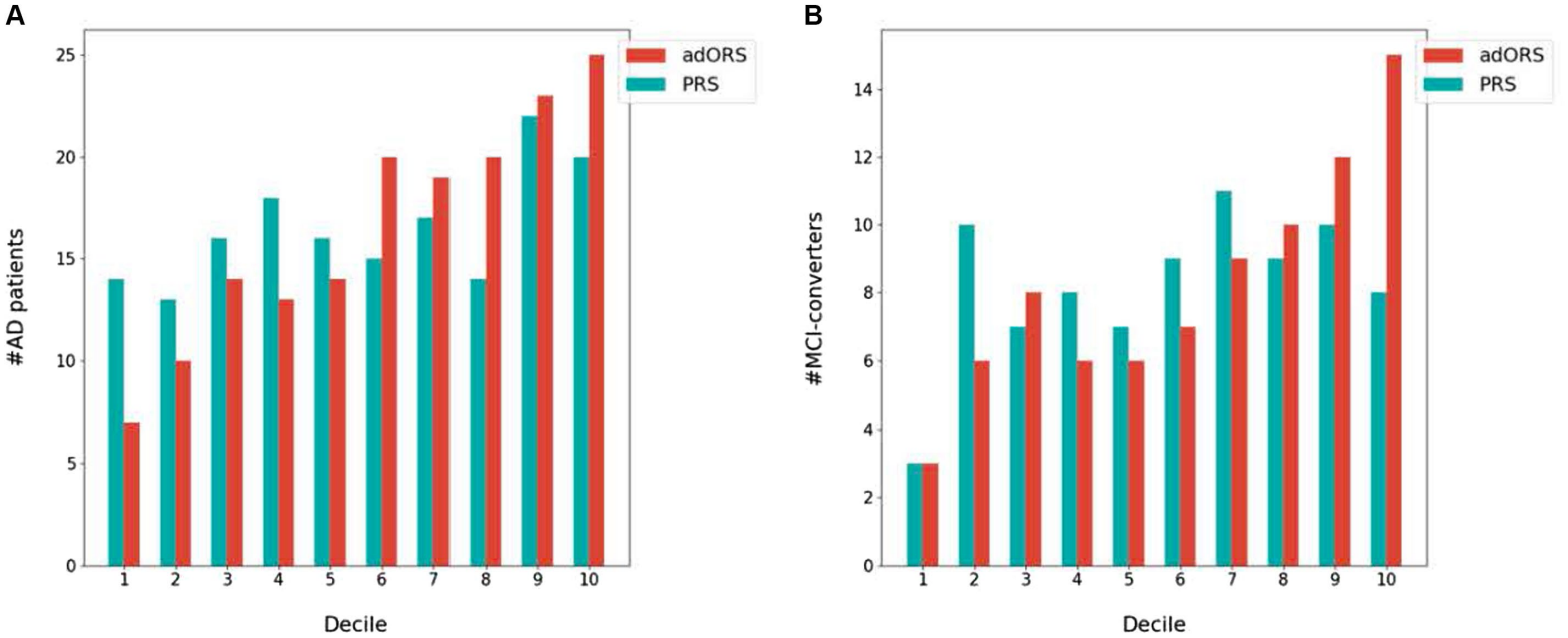
Stratification of AD cases and MCI converters into AD risk levels based on adORS.with.APOE and PRS.with.APOE. Decile analysis evaluating the ability of adORS.with.APOE and PRS.with.APOE to reflect the AD prevalence rate in **(A)** CN vs. AD classification and **(B)** MCI conversion prediction. The test set is evenly divided into deciles, or 10 bins, based on the adORS (red) or PRS (blue) values. Each bin contains the number of AD cases and MCI converters in **(A,B)**, respectively.

We next investigated each risk score’s ability to stratify the cohort into (Mullard, 2021) cases vs. controls and (Zetterberg and Bendlin, 2021) bins of increasing AD risk levels. We performed a decile analysis to evaluate how well each score truly represents the AD prevalence rate. Figure 1 presents the test set divided into 10 equally sized bins based on risk score values and the number of actual cases within each bin. In both the CNAD (Figure 1A) and MCI-CP (Figure 1B) tasks, adORS demonstrates a staircase effect from the first to last decile, indicating that the score is well-calibrated to risk level compared to PRS. As the adORS model assigns higher risk scores to patients, the number of cases in higher risk groups increases accordingly. However, the decile of risk scores binned by PRS appears less as a staircase effect and more uniform across all bins. This indicates that PRS hardly reflects the true prevalence rate within ADNI cohort. Similar trends are seen for the adORS and PRS models which do not include *APOE* (Supplementary Figure 2). Overall, adORS’s ability to improve stratification of patients into appropriate risk groups demonstrates that adORS is more well-calibrated to AD risk than PRS.

### 3.3. Gene contribution

**Figure 2:**
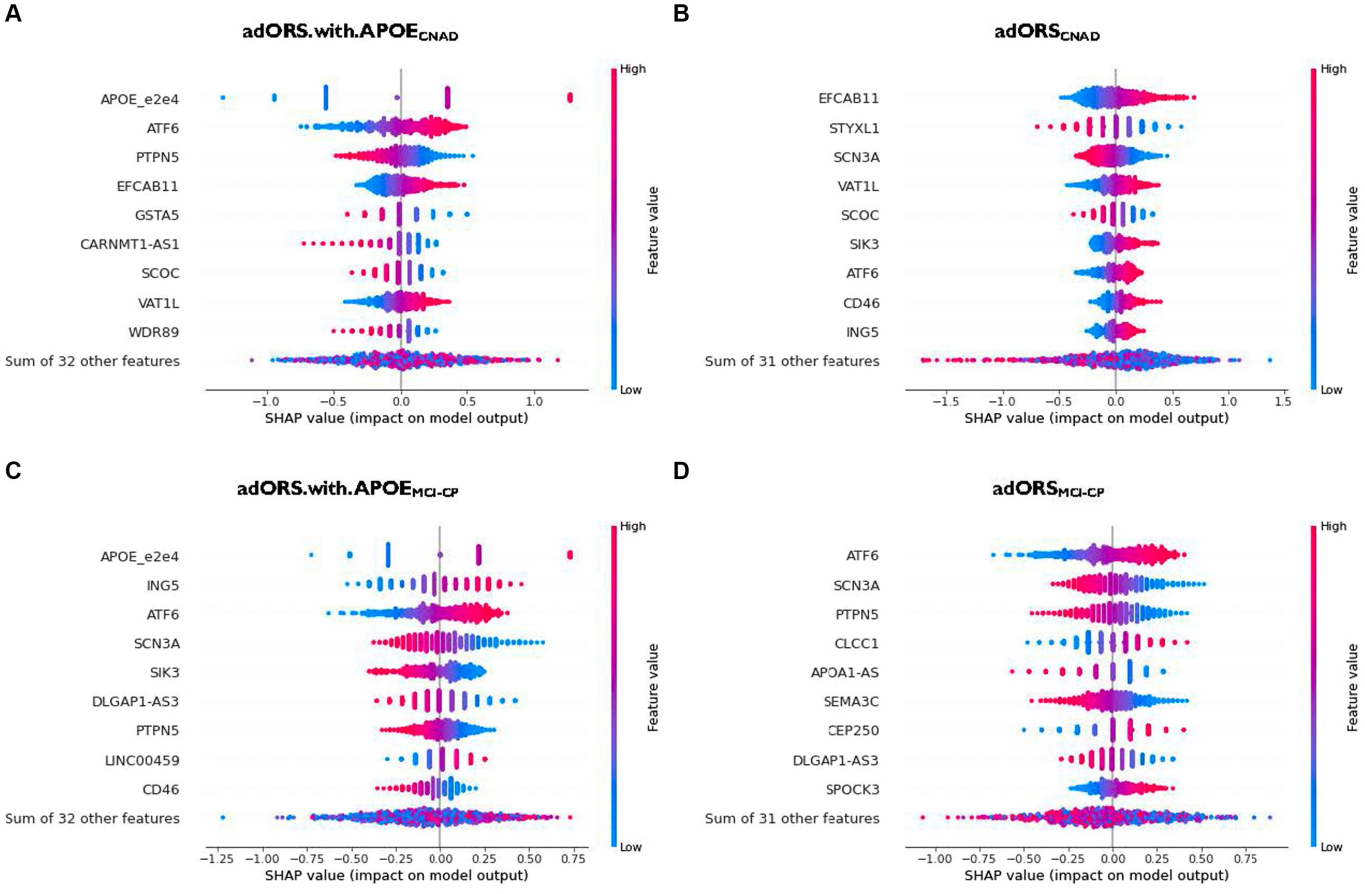
Gene contribution in adORS.with.APOE and adORS models. Beeswarm plots to visualize the gene-level contributions in adORS.with.APOE and adORS when performing CN vs AD classification **(A** and **B)** and MCl conversion prediction **(C** and **D)**. Patients are represented as a scatter plot in each row of each plot. The x-axis is determined by the SHAP value (gene contribution), and the color represents the gene burden value. The sign of the SHAP value represents the direction of contribution. For example, a negative SHAP value of a gene contributes to a decrease in risk score while a positive SHAP value contributes to an increase in risk score. See Supplementary Figure 3 for the gene contributions of PRS and PRS.with.APOE. CNAD, CN vs AD classification; MCI-CP, MCI conversion prediction.

The top contributors of adORS.with.APOE_CNAD_ and adORS_CNAD_ are *APOE* and EFCAB11, respectively, given that both genes demonstrate the highest magnitude of the SHAP value (Figures 2A,B; Supplementary Figures 3A,B). There is no overlap between the subsequent top five contributors across both models, excluding *EFCAB11*. This lack of overlap implies that the top genes found in adORS_CNAD_ may have associations with *APOE*, given that they are assumedly replacing *APOE*’s role. *APOE* also remains the top contributor for adORS.with.APOE_MCI-CP_ (Figure 2C; Supplementary Figure 3C). In adORS_MCI-CP_, *ATF6* is the top contributor, with *SCN3A* being the second-most contributor (Figure 2D; Supplementary Figure 3D). *ATF6* and *SCN3A* are also part of the top five contributors in adORS.with.APOE_MCI-CP_. There is very little overlap in gene contributions between the adORS and PRS models in both tasks (Figure 2; Supplementary Figures 3E–H), indicating that a different set of genes were considered to calculate PRS versus adORS.

As we reported in the *adORS* vs. *PRS model performance comparison* section, performance metrics suggested that PRS relies on *APOE* more so than in adORS, causing remainder variants to have low effects. As such, interpretation of genes—beyond *APOE*—that add to AD risk becomes more difficult for the PRS models. We further explored this notion using our gene contribution analysis, as shown in Supplementary Figure 3. The contribution of *APOE* in adORS.with.APOE_CNAD_ vs. PRS.with.APOE_CNAD_ is 19.6 and 20.9%, respectively (Supplementary Figures 3A,E). While these values are comparable, the distribution of contributions across the remainder of the genes is more dispersed in adORS.with.APOE_CNAD_. Most of the genes excluding *APOE* contribute about 2.3–7.2% to the prediction task (Supplementary Figure 3A), whereas in PRS.with.APOE_CNAD_, contributions range from 1.7 to 4.7%, with more than half of the genes contributing less than 1.7% (Supplementary Figure 3E). For MCI-CP, the difference is even more evident—*APOE* contributes 13.0% in adORS.with.APOE_MCI-CP_ and 62.5% in PRS.with.APOE_MCI-CP_ (Supplementary Figures 3C,G). The others contribute 2.2–6.9%, while in PRS, the top gene besides APOE contributes 2.3% at most, with nearly the rest contributing less than 1%.

We also investigated the direction of effect between the gene burdens and risk scores. In CN vs. AD classification, genes including *APOE*, *ATF6*, *EFCAB11*, *VAT1L*, *SIK3*, *CD46*, and *ING5* demonstrate a consistent, positive effect on the risk score (Figures 2A,B). Their SHAP values increase as their original values, or gene burdens, also increase. That is, higher gene burden values elicit higher risk for AD, and vice versa. In MCI conversion prediction, *ING5*, *ATF6*, *LINC00459*, *CLCCC1*, *CEP250*, and *SPOCK3* are observed to be positive contributors, while *SCN3A*, *SIK3*, *DLGAP1-AS3*, *PTPN5*, *CD46*, *APOA1-AS*, and *SEMA3C* are negative contributors (Figures 2C,D).

Interestingly, *SIK3* and *CD46* display an inconsistent direction of effect on the risk score across each task. *SIK3* in adORS.with.APOE_MCI-CP_, for example, exhibits a negative effect on the risk score, yet the effect is reversed in adORS_CNAD_ (Figures 2B,C). The same trends are observed for *CD46*. This reversal of effect from MCI-CP to CNAD suggests that both genes may play an important role in the progression of AD.

### 3.4. Phenotype association

**Figure 3:**
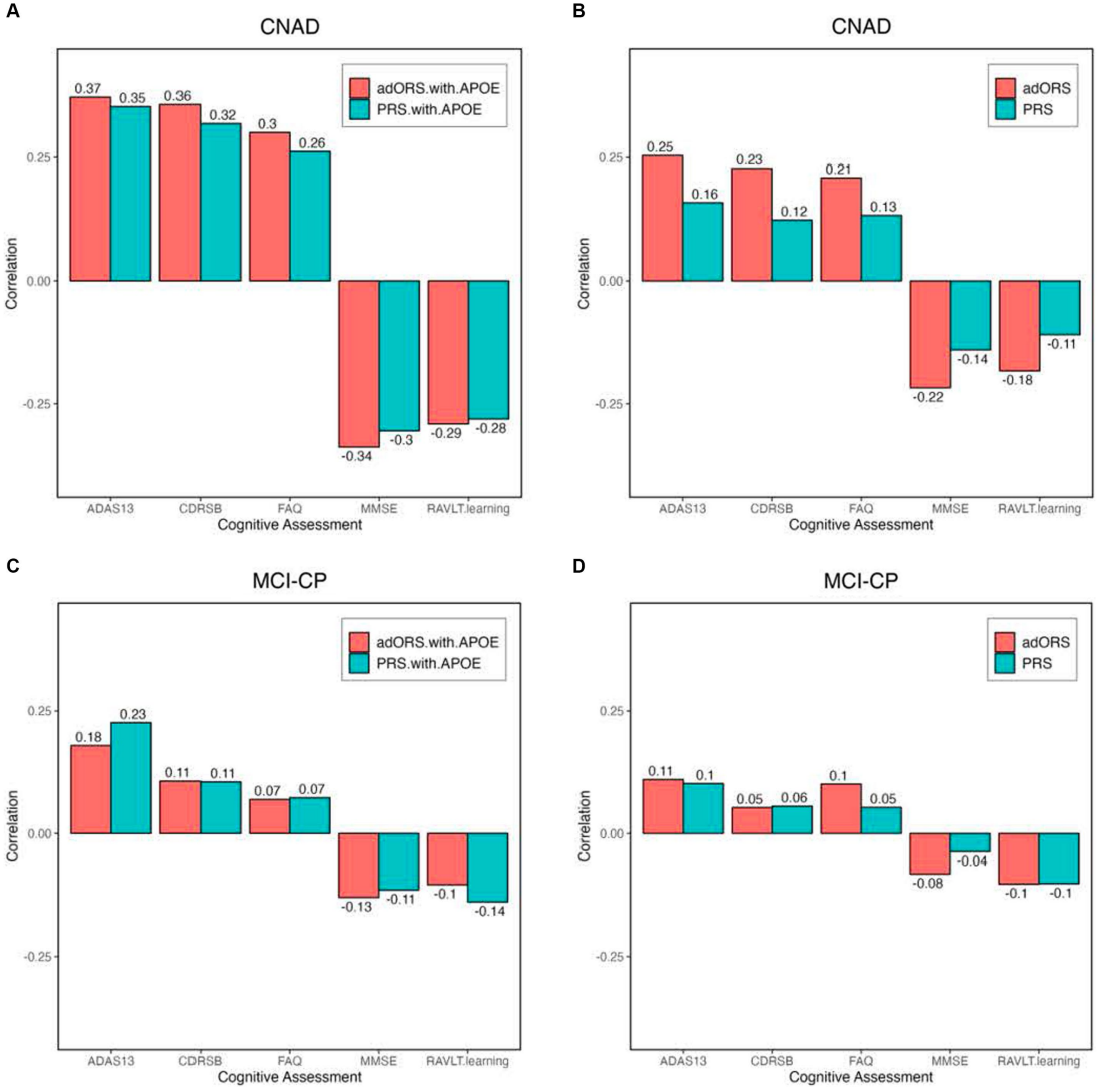
PRS, PRS.with.APOE, adORS, and adORS.with.APOE associations with major AD clinical assessments of cognitive functions. Bar plots of Pearson correlations between each risk score (adORS: red, PRS: blue) and cognitive assessments in the entire cohort. Results are shown for the following tasks: CN vs. AD classification **(A** and **B)** and MCI conversion prediction **(C** and **D)**. CNAD, CN vs. AD classification; MCI-CP, MCI conversion prediction; ADAS 13, Alzheimer’s disease assessment scale-cognitive subscale 13; CDRSB, Clinical dementia rating scale-sum of boxes; RAVLT, Rey auditory verbal learning test; MMSE, Mini mental state examination; and FAQ, Functional activities questionnaire.

We performed an association analysis on PRS and adORS with major AD clinical assessments of cognitive functions (ADAS13, CDRSB, RAVLT, MMSE, and FAQ; Portland Institute for Computational Science, 2022) widely used as evidence for AD diagnosis (Albert et al., 2011). We assessed the Pearson correlations between the estimated risk scores and these cognitive assessment scores (Figure 3). Both PRS_CNAD_ and adORS_CNAD_ appropriately showed a positive correlation with ADAS13/CDRSB/FAQ (Figure 3B). We also observed a negative correlation between the estimated risk scores and MMSE/RAVLT, which is as expected. Compared to PRS_CNAD_, adORS_CNAD_ had almost 2-fold higher absolute correlations. When *APOE* was included, adORS.with.APOE_CNAD_ still remained more strongly correlated than did PRS.with.APOE_CNAD_, yet the trends of PRS.with.APOE_CNAD_’s correlation values improve vastly in comparison to PRS_CNAD_ (Figure 3A). This evidence further supports the assumption that PRS heavily weights the *APOE* loci over other variants to sufficiently predict risk.

In general, MCI conversion prediction is more difficult than CN vs. AD classification. Correlations varied between adORS.with.APOE_MCI-CP_ and PRS.with.APOE_MCI-CP_, with adORS.with.APOE_MCI-CP_ performing better for MMSE while PRS.with.APOE_MCI-CP_ performed better for ADAS13/RAVLT (Figure 3C). This is consistent with the model performances shown in Table 3, where PRS.with.APOE_MCI-CP_ resulted in a higher AUROC than adORS.with.APOE_MCI-CP_. Without *APOE*, adORS_MCI-CP_ improved in comparison to PRS_MCI-CP_, with comparable yet better correlations in all cognitive categories except for CDRSB (Figure 3D).

## 4. Discussion

Given the lack of successful clinical trials for Alzheimer’s disease to date, the field has shifted some of its focus to genetic risk stratification using PRS in hopes to improve mechanistic insights of the disease (Sierksma et al., 2020). However, whether AD is a polygenic or oligogenic disorder remains unclear, and many suspect PRS’s ability to accurately identify high and low risk individuals, as well as effectively predict AD progression. In the current study, we generated adORS, an AD-specific oligogenic risk score that challenges the limitations of PRS in terms of prediction, stratification, and interpretability. Whereas PRS is derived from the weighted sum of GWAS-significant SNPs, adORS utilizes genes strongly associated with the neuroimaging biomarkers FDG-PET and AV45-PET to predict both AD and MCI conversion risk. The introduced score outperforms traditional PRS by providing enhanced prediction of AD risk. Additionally, adORS better stratifies individuals into increasing risk levels based on their genetic predispositions, reflecting the true prevalence rate of the cohort compared to PRS. Lastly, adORS delves into gene-level interpretations of the risk factors, paving the way for personalized therapeutic strategies for individuals with AD.

We conclude that the empirical data assessing AD risk are consistent with an oligogenic architecture of the disease. To represent oligogenicity of the disease, we implemented a gene-level burden analysis which collapses variants into genes. Because SNP data are extremely sparse and sample size is limited, we attempted to enlarge the gene-level effects that are relevant to AD by significantly reducing the feature size from 1.7 million SNPs to 23,000 genes. In other words, this approach allowed a denser input which permits the prediction model to train with increased stability. We expected several main effects from this architecture: enrichment of the AD-related signal, reduction of the degree of freedom, and gene-level interpretability. We anticipate that these outcomes may help establish the prospective clinical utility of adORS which PRS has been lacking thus far.

Compared to PRS, we found that the oligogenic model better represented the genetic heterogeneity of the disease by improving risk-based stratification for both CN vs. AD classification and MCI conversion prediction (Figure 1). When APOE was included in both models, the staircase effect portrayed in Figure 1 demonstrated that adORS is well-calibrated to AD risk level, whereas the decile of risk scores binned by PRS appears less as a staircase effect and more uniform across all bins. In other words, as adORS assigned higher risk scores to patients, the number of cases in higher risk groups increases accordingly. By refining stratification, adORS may prove to be a more appropriate choice of differentiating patients into high or low genetic risk of AD in clinical studies or settings. We were able to further support adORS’s potential clinical utility via our association analysis with common cognitive assessments used to clinically evaluate risk for AD (Figure 3).

However, while *APOE* is the most prominent genetic risk factor for AD, not all AD patients carry the APOE risk allele (Guerreiro et al., 2012). As such, we deemed it essential to conduct analyses both inclusive and exclusive of *APOE* effects. The conventional PRS model has been evidenced to heavily depend on *APOE* and nongenetic factors, such as age and sex, for effective risk assessment, which compromises its clinical relevance (Riedel et al., 2016; Harrison et al., 2020; Leonenko et al., 2021). When *APOE* effects were excluded from the adORS and PRS models, adORS still improved the stratification of patients from lowest to highest risk group, while PRS continued to disperse patients more uniformly across all risk levels (Supplementary Figure 2). Moreover, the overwhelming contribution of *APOE* in PRS made it challenging to gage the significance of individual SNPs or genes, especially in predicting MCI conversion (Supplementary Figures 3E,G). This disproportionate contribution by APOE obscures the roles of other genetic components, leaving us to question their significance in the overall risk model. adORS overcomes this limitation, as evidenced not only by the improved stratification results without *APOE*, but also by the gene-level interpretability analyses with and without *APOE*. Within the adORS framework, the genetic contributions are more balanced and dispersed, ensuring that no single gene, including *APOE*, monopolizes the risk prediction (Supplementary Figures 3A,C). This distribution not only provides a more holistic view of the multifactorial contributors to the disease, but also unmasks the latent potential of these genes in AD pathology. The ability of adORS to interpret specific genetic information allows the focus to be shifted from general relative risk based on a given population to the information that adORS can provide for a single individual, thus permitting the possibility of personalized treatments for AD.

As expected, *APOE* had the highest contribution in the adORS.with.APOE models for both CN vs. AD classification and MCI conversion prediction (Figures 2A,C; Supplementary Figures 3A,C). *APOE* pathogenesis is known to affect amyloid-β peptide aggregation and clearance, as well as tau neurofibrillary degeneration, microglia and astrocyte responses, and blood–brain barrier disruption (Serrano-Pozo et al., 2021). *ATF6* achieved the subsequent highest contribution in adORS.with.APOE_CNAD_ (Figure 2A; Supplementary Figure 3A). This gene was also the top contributor for adORS_MCI-CP_ (Figure 2D; Supplementary Figure 3D). In a recent study, *ATF6* demonstrated an involvement in reducing amyloid precursor protein expressions in AD model mice (Du et al., 2020). *ATF6* was also found to be strongly activated in AD and amyotrophic lateral sclerosis (ALS) individuals in pathways related to endoplasmic reticulum homeostasis (Montibeller and de Belleroche, 2018). *ING5*, a tumor suppressor protein that inhibits cell growth, achieved the second-highest contribution in adORS.with.APOE_MCI-CP_ (Figure 2C; Supplementary Figure 3C). *ING5* is associated with various cancer types and rare diseases involving neuronal abnormalities, such as Ohdo Syndrome (Gou et al., 2015; Zhang et al., 2015; Gene Cards Human Gene Database, 2022). In particular, *ING5* was found to be highly expressed in stem cells which promotes self-renewal of brain tumor initiating cells, which typically lead to glioblastomas (Wang et al., 2018). Another study has found that *ING5* is silenced by short hairpin RNAs during the generation of neuronal precursor cells (Zhang et al., 2013). The evidence of *ING5* in neuronal-related development suggests a possible connection to AD pathophysiology.

In adORS_CNAD_, *EFCAB11*, which is predicted to enable calcium binding activity, was the highest contributing factor for AD classification (Figure 2B; Supplementary Figure 3B). Circular RNAs (circRNAs), including *EFCAB11*, have been found to be highly expressed in the brain compared to other tissues, suggesting that circRNAs may constitute as relevant aging biomarkers (Pan et al., 2020). One research group found *EFCAB11* to be overexpressed in brain samples of patients with multiple system atrophy, another type of neurodegenerative disorder (Chen et al., 2016). Although there is little information specific to *EFCAB11* in AD, studies have found connections of alternative circRNAs to AD, such as dysregulated cIRS-7-miR-7 interaction in the hippocampus of AD patients (Lukiw, 2013; Zhao et al., 2022). Such evidence proposes circRNAs as potential targets for AD treatment.

Other genes observed in the various adORS models and supported by AD-related literature include *PTPN5* and *VAT1L*. *PTPN5* (seen in Figure 2; Supplementary Figures 3A–D) is involved in pathways that regulate neuronal signal transduction, neuronal maturation and survival, synaptic function, and learning and memory. Abnormal alterations in *PTPN5* activity are associated with several neurological disorders, and it is known that upregulation of *PTPN5* may have a causal role in Alzheimer’s disease (Chin et al., 2005; Saavedra et al., 2011). *VAT1L* (seen in Figures 2A,B; Supplementary Figures 3A,B) has been identified as associated with functions related to neuronal maintenance, neurotransmission, and Tau pathology (Jain et al., 1991; Dringen, 2000).

*SIK3* and *CD46* displayed reversed directions of effect from adORS.with.APOE_MCI-CP_ to adORS_CNAD_ (Figures 2B,C). In MCI-CP, lower gene burden values for both genes resulted in higher risk scores, whereas this effect was reversed in CNAD. Limited investigation exists on *SIK3*’s role in AD. A gain-of-function *SIK3* mutation has previously been shown to cause an acute decrease in total wake time, resulting in high sleep need despite increased sleep amount (Funato et al., 2016; Wang et al., 2018; Zhou et al., 2022). Interestingly, literature also evidences that sleep disturbances occur more frequently as AD increases in severity. Excessive daytime sleepiness, one of the most common sleep disturbances in demented patients, is characterized by symptoms that align with the *SIK3* mutation, as mentioned above (Vitiello et al., 1990; Honda et al., 2018; Wang and Holtzman, 2020). Further research linking *SIK3*-involved pathways, sleep regulation, and AD may provide additional insight in AD pathology. Similar to *SIK3*, *CD46* is also rarely discussed in relation to AD. *CD46* is a significant complement receptor protein that promotes human cytotoxic *CD8*+ T cell activity and relates with autoimmune diseases (Liszewski and Atkinson, 2015; Arbore et al., 2018; Stein et al., 2019). One study identified *CD46* as significantly dysregulated in AD patients based on a transcriptome-wide meta-analysis of blood-based microarray gene expression profiles (Nho et al., 2020). The complement system has also been previously linked to AD. An AD-protective variant of *CD33*—another complement protein—is postulated to truncate the protein, reducing functions such as cell signaling and inflammation (Siddiqui et al., 2017). This suggests that lower expression of complement genes may have beneficial effects on the brain, supporting our data that higher *CD46* gene burden values resulted in higher risk scores in the CNAD task. The reversal of effects for both genes may indicate a potential shift in their roles within MCI progression to AD. We suggest further research exploring the effects of *SIK3* and *CD46* particularly in MCI converter patients.

In clinical settings, adORS can be particularly valuable for trials targeting therapies to prevent disease progression. By identifying cognitively normal adults at high AD risk through an elevated adORS, clinicians can concentrate on those most representative of disease progression and most likely to benefit. This approach also enhances trial efficiency and cost-effectiveness (Lewis and Vassos, 2020). adORS also has the potential to aid treatment decisions. For example, individuals with a low adORS might postpone screenings, whereas those with a higher score could start earlier than typically advised (Lewis and Vassos, 2020). Especially for a condition such as AD, in which treatments target symptom management, pinpointing individuals at high genetic risk for earlier interventions can elevate their quality of life. However, it is crucial to note that adORS merely indicates a genetic predisposition to the disease and cannot serve as a diagnosis. As risk scores gain broader applications in both clinical and research settings, recognizing the difference between prediction and definitive diagnosis is essential.

There are several limitations in this study. Note that the success of a gene-level burden analysis depends on several conditions (Li and Leal, 2008). First, the model input should be limited to functional variants and exclude nonfunctional variants. Restricting input to such is crucial to building an interpretable and well-generalized model that performs well across multiple test cohorts. Currently, our model collapses both functional and nonfunctional variants into genes, which likely introduces noise and thus hinders our model from detecting relevant genes with increased accuracy. Although aggregation methods can amplify association signals and minimize the degrees of freedom more effectively than variant-level tests, they are not always robust to the misclassification of nonfunctional variants. We are actively exploring this avenue in our ongoing research, in which nonfunctional variants are separately considered and grouped according to their intergenic regions. Second, we assume that the direction of effect is consistent within genes. Indeed, two variants with opposite directions of effect will be canceled out. While we adjust for variants negatively associated with AD through the complement operation (see section 2.5), it is also vital to weigh the magnitude and statistical relevance of these effects. As long as we implement a gene-level burden analysis, information loss is inevitable due to the inclusion of nonfunctional variants and exclusion of functional variants. Enhancing the representation of nonfunctional variants is expected to improve the prediction performance. We are investigating the possibility as a sequel to this work.

To ensure generalizability, adORS must be validated on external cohorts. The datasets suitable for training are somewhat restrictive, as both genomic and neuroimaging data must be available to compute our proposed risk score. Despite the constrained number of training data, our model still demonstrates improved performance. Gathering larger training sets is likely to produce a significant boost in accuracy. For validation purposes, adORS has the benefit of requiring only genomic data. For example, by leveraging the adORS generated using ADNI, we can extend its application to other sizable cohorts with available genomic data, such as the Alzheimer’s Disease Sequencing Project (ADSP) (2020), and evaluate its proficiency in stratifying the population based on risk levels. Among the many neuroimaging biomarkers that are available in the ADNI dataset, only two were used to generate adORS. In doing so, we may have inadvertently excluded SNPs which are highly associated with other AD biomarkers and may therefore hold predictive ability for AD risk. Additionally, this score only relies on common variants and excludes rare variants. We are considering these limitations as well in the continuations of this study.

Overall, we developed adORS to leverage genetic information that can identify individuals with high or low risk of developing Alzheimer’s disease. Compared to conventional PRS, adORS was able to improve prediction performance, risk-based stratification of patients, and interpretability of genetic factors contributing to risk. Further study is required to functionally validate the genes in adORS and their roles in underlying AD-related processes, and ultimately contribute to risk or progression-related biomarkers that can aid in the downstream development of therapeutic treatments for AD.

## Data availability statement

The original contributions presented in the study are included in the article/Supplementary material. Further inquiries can be directed to the corresponding author.

## Ethics statement

The ADNI study was approved by the individual Institutional Review Boards of all participating institutions, with written informed consent obtained from all participants or their guardians according to the Declaration of Helsinki (consent for research). All data were deidentified.

## Author contributions

ES: Conceptualization, Formal analysis, Investigation, Methodology, Validation, Visualization, Writing – original draft, Writing – review & editing. GL: Conceptualization, Formal analysis, Investigation, Methodology, Validation, Visualization, Writing – original draft, Writing – review & editing. S-HJ: Formal analysis, Validation, Writing – original draft, Writing – review & editing. ZW: Formal analysis, Writing – original draft, Writing – review & editing. JB: Formal analysis, Writing – original draft, Writing – review & editing. KN: Resources, Writing – review & editing. HH: Resources, Writing – review & editing. CD: Resources, Writing – review & editing. AS: Resources, Writing – review & editing. PT: Resources, Writing – review & editing. LS: Resources, Writing – review & editing. DK: Conceptualization, Funding acquisition, Resources, Supervision, Writing – review & editing.
