# Supplementary material for: An interpretable Alzheimer’s disease oligogenic risk score informed by neuroimaging biomarkers improves risk prediction and stratification
Source: Front Aging Neurosci. 2023 Oct 26;15:1281748. doi: 10.3389/fnagi.2023.1281748 (PMC10637854; doi:10.3389/fnagi.2023.1281748)
Supplement: Supplementary file 1 [file Data_Sheet_1.docx]

Supplementary Material

An interpretable Alzheimer’s disease oligogenic risk score informed by neuroimaging biomarkers improves risk prediction and stratification

**Erica H. Suh^1†^, Garam Lee^1,2†^, Sang-Hyuk Jung^1^, Zixuan Wen^1^, Jingxuan Bao^1^, Kwangsik Nho^3^, Heng Huang^4^, Christos Davatzikos^5^, Andrew J. Saykin^3^, Paul M. Thompson^6^, Li Shen^1,7^, and Dokyoon Kim^1,7*^ for the Alzheimer’s Disease Neuroimaging Initiative**

*** Correspondence:** Dokyoon Kim: dokyoon.kim@pennmedicine.upenn.edu

# ADNI Data Use

Data used in preparation of this article were obtained from the Alzheimer’s Disease Neuroimaging Initiative (ADNI) database (adni.loni.usc.edu). As such, the investigators within the ADNI contributed to the design and implementation of ADNI and/or provided data but did not participate in analysis or writing of this report. A complete listing of ADNI investigators can be found at: <http://adni.loni.usc.edu/wp-content/uploads/how_to_apply/ADNI_Acknowledgement_List.pdf>

# Supplementary Figures and Tables

# Each figure is placed on its own page below. Refer to the excel sheet for supplementary tables.


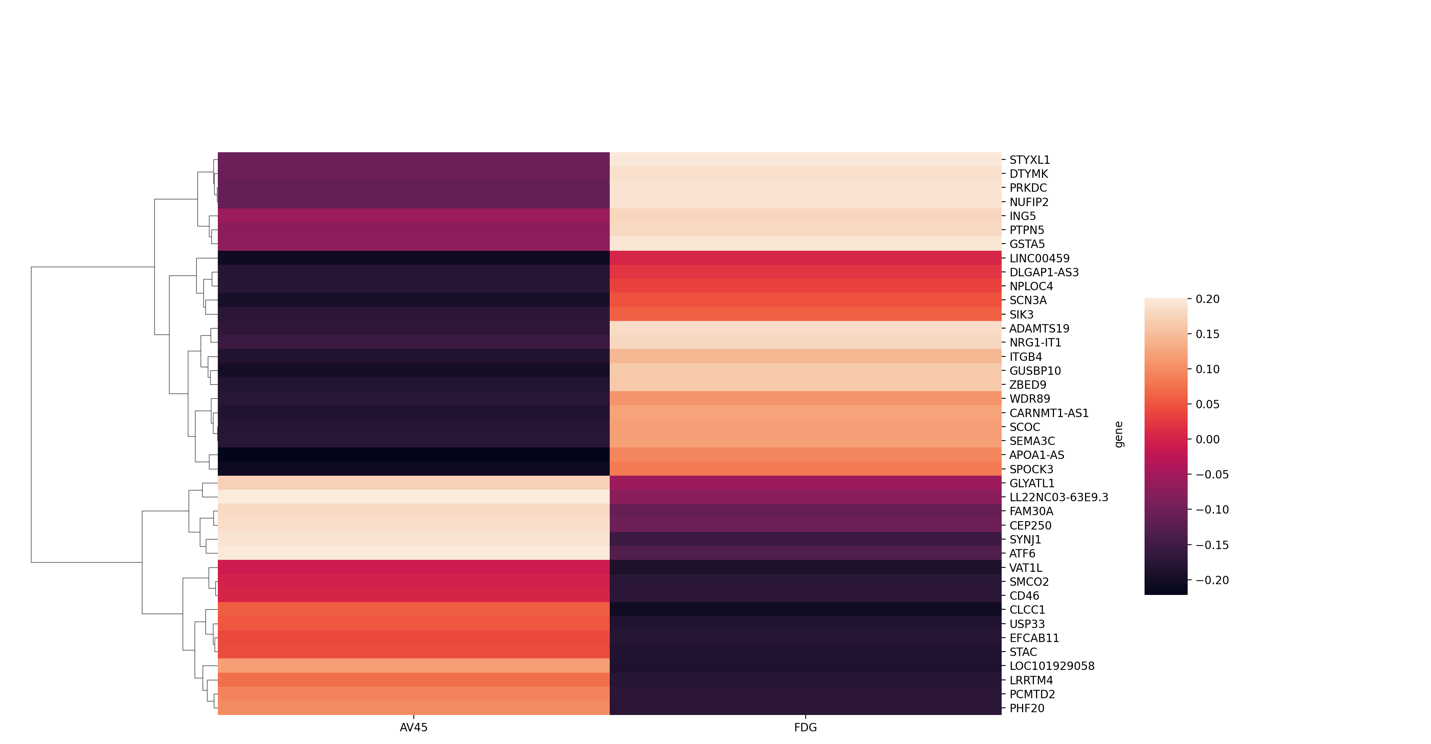


**Supplementary Figure 1 Pearson *c*orrelation matrix between selected genes and neuroimaging biomarkers AV45-PET and FDG-PET.** Correlation heatmap of each of the top 20 genes that have the highest Pearson correlations with neuroimaging biomarkers AV45-PET and FDG-PET. Genes specified in this plot are those that are included in adORS calculation (see *adORS calculation* in Methods and **Supplementary Table 2**).

1. **
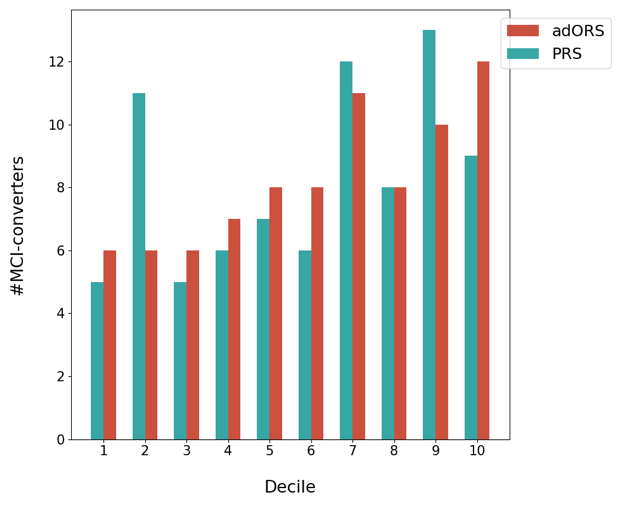

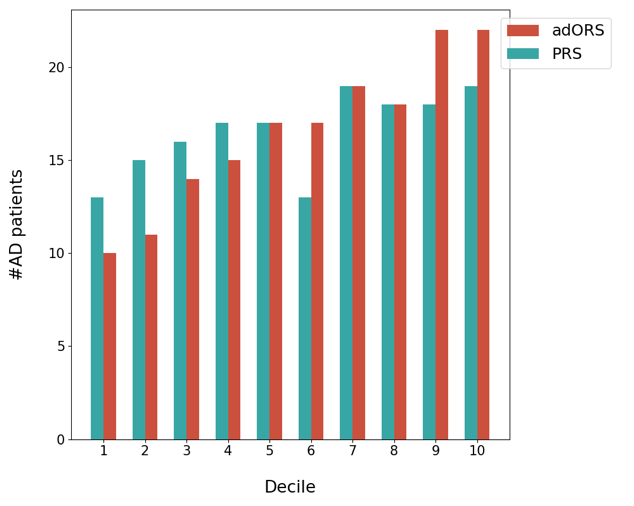
**  **B)**

**Supplementary Figure 2 Stratification of AD cases and MCI converters into AD risk levels based on adORS and PRS.** Decile analysis evaluating the ability of adORS and PRS (both models without APOE) to reflect the AD prevalence rate in A) CN vs AD classification and B) MCI conversion prediction. The test set is evenly divided into deciles, or ten bins, based on the adORS (red) or PRS (blue) values. Each bin contains the number of AD cases and MCI converters in A and B respectively.

## Supplementary Figure 3 Gene contribution percentages in adORS and PRS models.

| **adORS models** | |
| --- | --- |
| 1. adORS.with.APOE_CNAD_   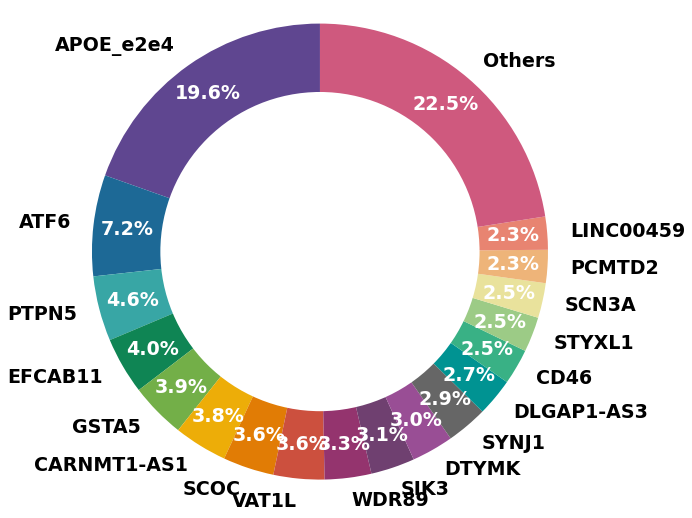 | 1. adORS_CNAD_   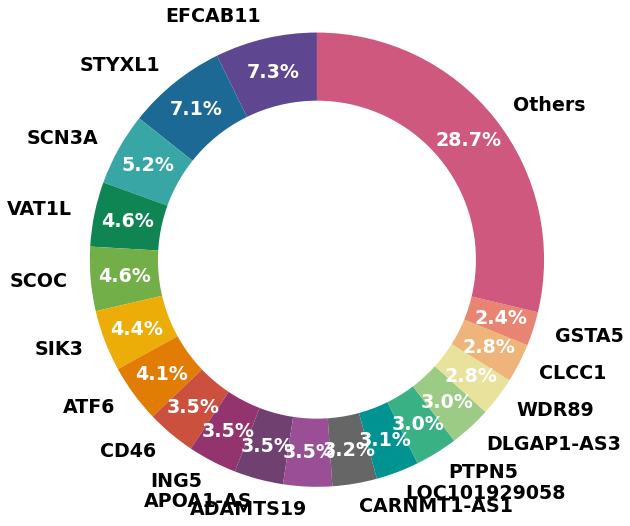 |
| 1. adORS.with.APOE­_MCI-CP_   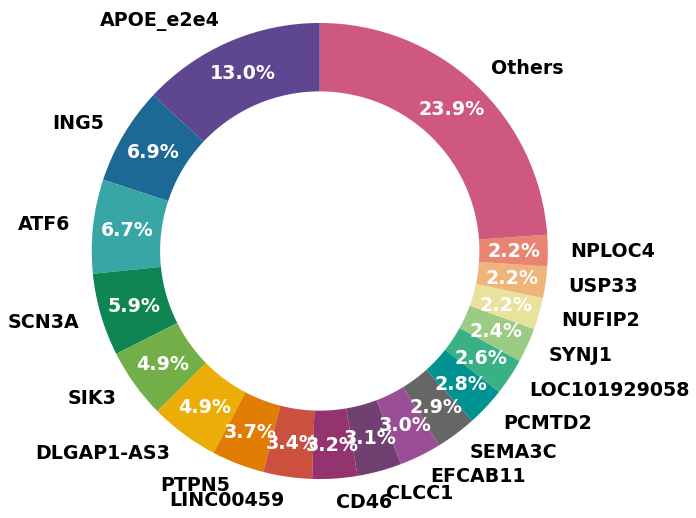 | 1. adORS_MCI-CP_   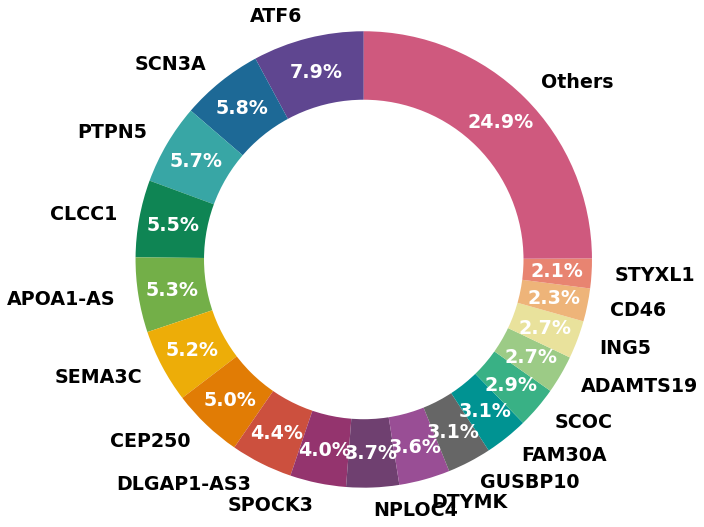 |

**Supplementary Figure 3 continues onto next page*

**Supplementary Figure 3** *Continued from previous page*

| **PRS models** | |
| --- | --- |
| 1. PRS.with.APOE_CNAD_   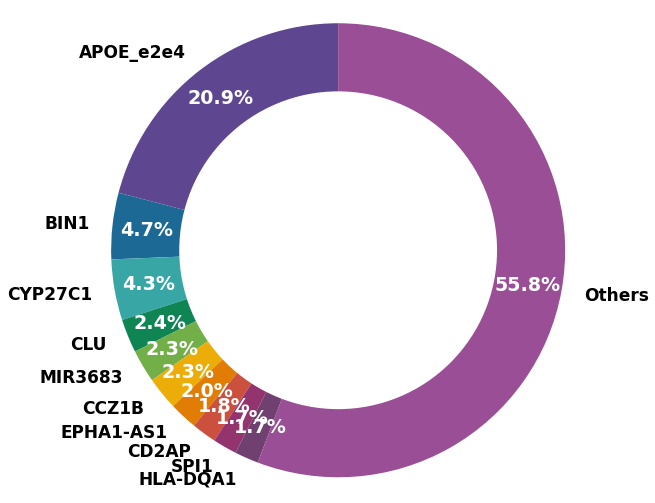 | 1. PRS_CNAD_   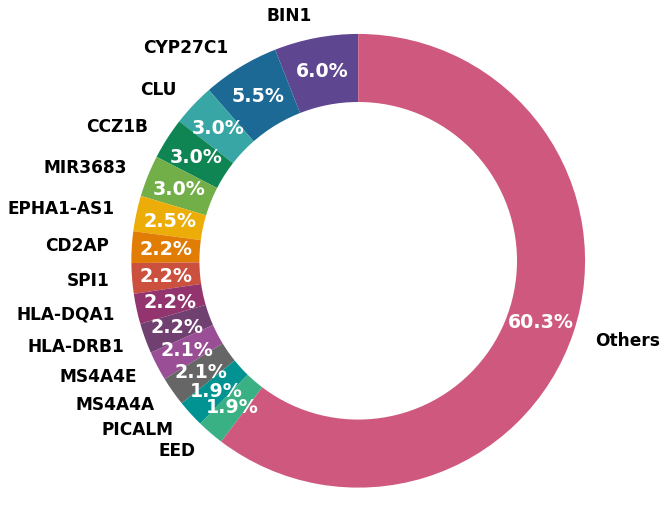 |
| 1. PRS.with.APOE_MCI-CP_   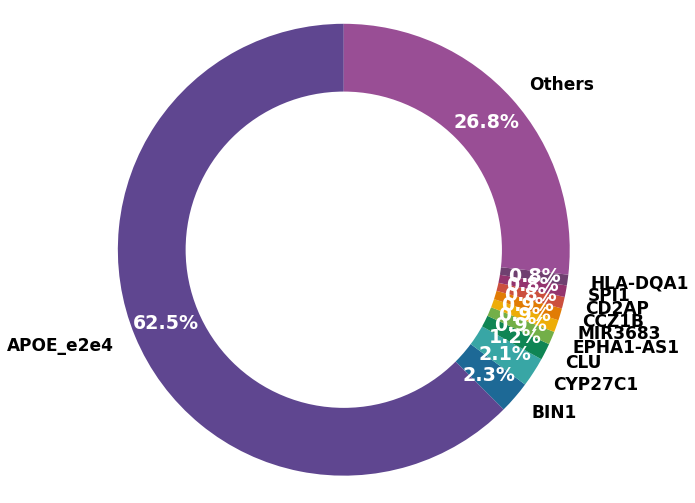 | 1. PRS_MCI-CP_   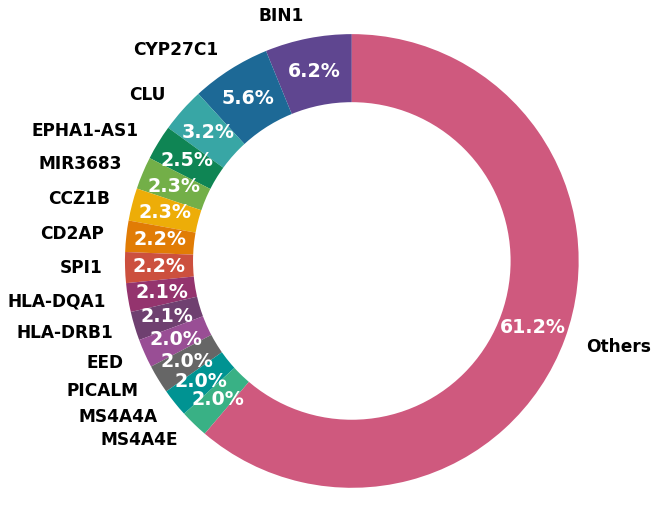 |

## Supplementary Figure 3 Gene contribution percentages in adORS and PRS models. Pie chart of the genes incorporated in each model and their percentages of contribution in performing CN vs AD classification (top row of each section) and MCI conversion prediction (bottom row of each section). PRS and PRS.with.APOE include SNPs with a p-value threshold of 1e-5. CNAD: CN vs AD classification, MCI-CP: MCI conversion prediction.
